# Responsible Business Conduct in Commodity Trading—A Multidisciplinary Review

**DOI:** 10.1007/s10551-024-05635-w

**Published:** 2024-03-27

**Authors:** Henrietta Dorfmüller, Wangui Kimotho, Isabel Ebert, Pascal Dey, Florian Wettstein

**Affiliations:** 1 University of St. Gallen, St. Gallen, Switzerland; 2 Bern University of Applied Sciences, Bern, Switzerland

**Keywords:** Commodity trading, Responsible business conduct, Global value chains, Human rights, Environment, Sustainability, Literature review, Multidisciplinarity

## Abstract

Responsible business conduct (RBC)—the corporate activities and initiatives that proactively address corporate involvement in human rights, environmental, and governance threats—has become an increasingly used means to counteract and prevent adverse effects of global businesses. Unlike other business sectors whose adverse impacts and RBC efforts (or lack thereof) are well documented, a comprehensive understanding of the state of commodity trading (CT), has been missing. In response, this paper uses a multidisciplinary literature review to provide an integrative understanding of the current state of research on the relationship between CT and RBC. Based on a review of 131 articles, we advance a granular understanding of the current and prospective role of commodity traders in RBC by grouping extant research into three overarching themes: (1) industry self-regulation and co-regulatory initiatives, (2) government-led regulatory initiatives and policy responses, and (3) company-level management strategies impacting RBC practices. In addition to illustrating the themes through existing research and identifying gaps along the overarching themes, we use our literature review to suggest avenues for future research. The paper’s overarching contribution is, first, to synthesize previously fragmented findings into a coherent framework of CT and RBC. And second, to offer guidance on how scholarship in this important domain can be developed into a more mature, legitimate and practical stream of research.

## Introduction

Commodity trading (CT) is integral and essential to global trade (Dejung, 2018). It is commonly agreed that commodity traders play an indispensable role in rendering global value chains (GVCs) efficient and effective by helping primary producers concentrate on their core business (Pirrong, 2014), while ensuring that manufacturers access raw materials to produce consumer and other related goods (Gilbert, 2008). Gibbon (2001) opines that GVCs are not less controlled and driven by producers or buyers than by international trading companies that play “a coordinative role in these commodity chains by virtue of being able to procure continuously specific volumes and quality mixes for a number of processers” (p. 351). Given their purported benefits, commodity traders are viewed by many as invaluable intermediaries driving the integration of the global economy (Baines & Hager, 2021). More skeptical assessments have been voiced by criticial commentators who have expressed concern about the dominant role of CT companies in GVCs, coupled with their often questionable business practices and adverse social (including human rights) and environmental footprint, as well as their limited degree of responsible business conduct (RBC) (Bürgi Bonanomi, et al., 2015). Indeed, while the globalized trade of commodities has resulted in increased wealth and prosperity for some countries and market actors, reports by international and non-governmental organizations suggest that CT is also linked to adverse environmental and social impacts (Public Eye, 2019, 2022; SOMO, 2020). In light of these controversial issues, it is striking that limited scholarly attention has been devoted to CT in its relationship to RBC (Baines & Hager, 2021; Dobler & Kesselring, 2019; Elsby, 2020). This holds not least for the dedicated literature on business ethics, which has traditionally had a strong focus on topics and perspectives relating to RBC, but has so far paid scant attention to CT (Hendrickson & James, 2005; Hira & Ferrie, 2006; Hofmann et al., 2018; Valkila et al., 2010). Consequently, as a comprehensive understanding of the relationship between CT and RBC is missing, this implies the need for an integrative approach that allows to determine the current state of knowledge on CT (and RBC), the gaps that exist in the literature, its relevance to business ethics, and issues that deserve scholarly attention in the future.

It is against this backdrop that we provide an integrative literature review of the current state of research on the intersection of CT and RBC. The relevance of our review is crucially linked to the recognition and importance of commodity traders in GVCs (Gibbon, 2001), the increasing ethical concerns expressed in regard to their negative spill-over effects on society and the environment (Dobler & Kesselring, 2019), and the associated need to steer CT toward ethical conduct. While a review approach seems well positioned to elevate CT and RBC to a place of central concern in management research, and business ethics more specifically, we consider a dedicated focus on CT and RBC to be timely and important for the following reasons: first, CT has a very broad scope, as it is closely linked to many economic areas (industries, branches, sectors) and, ultimately, everyday life, where it meets the growing demand for, among others, input materials for large infrastructure projects, food to feed a growing world population, and minerals and metals for various products. In this way, CT is an invaluable intermediary ensuring the smooth functioning of the global economy. Second, and conversely, CT is a highly contentious area of business activity (King & Pearce, 2010; Soule, 2009), as exemplified by CT’s frequently reported association with controversial practices such as financialization, bribery and illicit financial flows, and money laundering (Clapp, 2014; Clapp & Isakson, 2018). Third, CT has attracted relatively little attention as an empirical phenomenon and a subject of critical debate in management and business ethics research. A cursory look at the broader literature on responsible GVCs and global business confirms that extant research is focused primarily on upstream actors, such as producers (Lee et al., 2010), or downstream actors, such as manufacturers or retailers (Bair & Palpacuer, 2015; Durand & Jacqueminet, 2015; Elder & Dauvergne, 2015; Filatotchev & Stahl, 2015), whereas midstream actors such as commodity trading companies have received relatively little attention. Adding to this, there is a significant gap when it comes to discussing CT from a business ethics perspective, with only a very nascent discourse having started in the recent years (Hendrickson & James, 2005; Hira & Ferrie, 2006; Hofmann et al., 2018; Valkila et al., 2010). As a result, business ethics scholarship has not sufficiently outlined or theorized the inner workings and broader implications of CT practices, nor reflected on their broader relevance for business ethics. Furthermore, a conspicuous aspect of extant research on CT (and RBC), and a major reason why knowledge accumulation in this subject area has been slow, is that it is scattered across disciplinary silos. That is, while scholarly attention to CT has noticeably increased over the last years, leading to exciting new insights, research has remained fragmented as scholars only rarely incorporate or build on insights from other (sub-)disciplines.

By implication of these points, a literature review with a dedicated focus on CT is well suited to advance our understanding of arguably one of the most influential and controversial players in the commodity value chain. It is anchored in a multidisciplinary tradition that allows us to ‘break down’ existing silos (Jones & Gatrell, 2014) by analysing the current literature across disciplinary boundaries (Dekkers et al., 2022). The objective and contribution of our review is to: (1) analyze the current state of research on CT both in isolation (i.e., as a stand-alone actor) as well as its relationship with other value chain actors in the context of social and environmental impacts along the value chain; (2) provide an integrative view of the literature on RBC in CT, which is currently fragmented and scattered across different academic disciplines; and (3) sketch some promising avenues for future research, with a particular focus on how commodity traders, through their intermediary role, can adopt a more prominent role in anchoring RBC in GVCs.

This review article is structured as follows. We first define our key terms (i.e., commodities, paper and physical trade, and RBC) and conceptualize CT in its relationship with RBC. Second, we introduce the methodology, including the sampling procedure, the multidisciplinary design of our review and the time scope of our analysis. Third is a discussion of findings in two parts. The first part conveys descriptive information of the analyzed literature (publication chronology, and focus of articles in terms of (a) disciplinary and paradigmatic orientation, (b) commodity types, (c) value chain segments, and (d) RBC issues). The second part discusses the three overarching research themes that we identified: (1) industry self-regulation and co-regulatory initiatives, (2) government-led regulatory initiatives and policy responses on RBC in commodity trading, and (3) company-level management strategies. Fourth, we outline potential avenues for future research, specifically designed for scholars within the domains of management and business ethics. This is succeeded by a brief conclusion.

## Commodity Trading Along Global Value Chains

This section sets the scope of the literature review by defining the key concepts informing our review: commodities, paper and physical trade, and RBC. To begin with, commodities are broadly defined as any primary good that can be traded. These primary goods (as opposed to manufactured products) are (relatively) homogenous natural resources, which are traded in large volumes at a uniform price (WTO, 2010). Commodities are often classified into two broad categories: *soft commodities*, such as grains, cocoa, coffee, and livestock, and so-called *hard commodities,* which are extracted or mined and which include energy products like oil, natural gas, and coal, as well as metals and minerals such as gold, silver, aluminum, and cobalt.

**Fig. 1:**
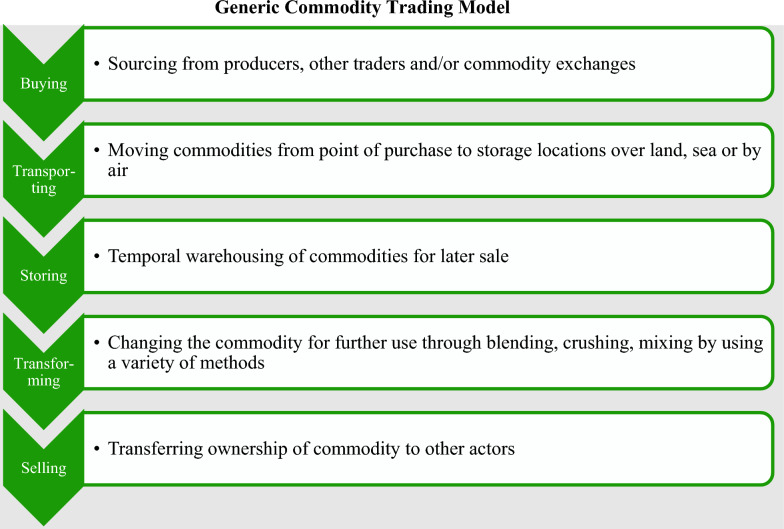
Generic Commodity Trading Model (Own illustration, adapted from Swiss Federal Department of Foreign Affairs & Swiss State Secretariat for Economic Affairs, 2018, p. 7)

Within this range of commodities, we can distinguish two types of trade: ‘paper trade’ and ‘physical trade’. In paper trade, transactions are based on “futures contracts (*of defined commodities*) [which are used to] specify a future price at a site of delivery” (Jacobs, 2014, p. 485) without commodities’ physical possession. The main market participants of paper trade are banks, investment funds, and institutional investors. Paper trade predominantly treats commodities as investment, risk-management (hedging), and speculation objects for which those involved aim to respectively achieve financial gains from or protect against commodity price changes (Clapp & Helleiner, 2012). Hedging of commercial risks is a crucial strategy for reducing traders’ exposure to flat^1^ commodity prices (Pirrong, 2014). Hedging the flat price risk results in a smaller and better manageable price risk for a CT company, albeit the risk is not completely eliminated (Pirrong, 2014). Physical trade, on the other hand, refers to the exchange of physical commodities through a series of generic activities (detailed in Fig. 1) that facilitate the flow of primary goods along the value chain (Pirrong, 2015).

After clarifying the general difference between paper and physical trade, three additional points deserve mention here. First, paper and physical trade, though analytically distinct, might overlap in practice (Baines, 2017; da Silva et al., 2003). Consider, as an example, a situation where commodity traders seek to limit price risks by using futures trading as a hedging tool (Pirrong, 2015; Salerno, 2017). This example helps us remind ourselves that both types of trade might be intricately interwoven in practice, although it might be challenging to determine the precise extent of their mutual interaction (Yang et al., 2005). Second, Baines (2017) reminds us that there might be a causal relationship between specific forms of paper trading (speculation) and price volatility, with traders potentially making large profits while producers such as small-scale farmers bear the negative impacts and risks (planning uncertainty and low and volatile prices) of speculation. Our main interest in this paper is in commodity traders’ core business, which, as we have argued, is the physical trading of commodities. Hence, our review includes articles that discuss paper trading only as a means of risk mitigation (i.e., hedging), and thus as an instrument in direct support of physical trade (Clapp, 2014; da Silva et al., 2003). Concordantly, we have not included articles on speculation in CT without any apparent link to physical trading. In this way, we deliberately exclude articles that exclusively deal with the financial side of CT. Third, over the last decade, there has been an increasing trend among the bigger trading companies toward vertical integration (backward and forward) by acquiring primary production facilities, logistics assets, such as tank storage and warehousing (Dobler & Kesselring, 2019), as well as processing and refining facilities (Fold, 2002; Ingram et al., 2018). Acknowledging that vertical integration has fundamentally confounded the conventional modus operandi and identity of CT, our review encompasses articles on vertically granted business models. However, CT remains heterogenous in terms of the scope and size of the companies involved (Pirrong, 2015).

We next define the concept of RBC. Broadly, RBC encompasses the myriad of corporate activities and initiatives, which try to increase and ensure a responsible response to the threats to human rights, environment, and governance related to their operations. The first discussions on RBC emerged in the 1950s (Bowen, 1953; Davis, 1960; Frederick, 1960; Votaw, 1961), although more systematic and elaborate debates only took place in the 1970s (Carroll, 1977) and 1980s (Donaldson, 1982). Business ethics scholarship, in particular, became the catalyst and ‘home’ for such debates during this period and has remained central to the theorization of RBC ever since. While early discussions dealt with fundamental questions concerning companies’ moral agency and the nature of their respective responsibilities (Donaldson, 1982; French, 1979; Werhane, 1989), they have become more granular and specific as time went on, covering a growing range of issues and spanning various disciplines. Hence, while academic discussions on the moral status of corporations as bearers of responsibility have continued to this day (e.g., Rönnegard, 2015), it is, by now, widely accepted both in practice and in theory, that corporations can be ascribed moral and social responsibility. Despite this general consensus, there is ongoing discussion and considerable controversy about how extensive such responsibility ought to be, with respective discussions essentially crystallizing around the differences between more narrow shareholder-centered and wider stakeholder-oriented conceptions of the firm (Gibson, 2000). While the former camp argues that corporations have a sole, or at least primary, fiduciary duty toward their shareholders to maximize returns, representatives of the latter defend varying more expansive notions of corporate responsibility beyond a privileged position of shareholders (Rose, 2007). While it would be beyond the scope of this paper to thoroughly engage in the normative justification of either of these positions, it shall suffice to mention that our interest in this paper is in the adoption of more expansive conceptions of RBC in the literature on CT, while fully acknowledging the normative contestation around such notions. In fact, the very observation that more traditional, shareholder- and profit-centered perspectives on the firm may still dominate both CT practice and literature, has been an important impetus for the focus of this analysis and its general relevance to the broader discussion and literature on business ethics.

Contemporary discussions on such more expansive ideas of RBC are clustered under various labels, such as corporate social responsibility (CSR), corporate citizenship, corporate sustainability, creating shared value, or corporate social performance, among others (Akbari & McClelland, 2020). In this sense, RBC is an umbrella term, which encompasses various contributions and foci, including normative depictions and philosophical justifications of corporate responsibility (e.g., Goodpaster, 2007; Ulrich, 2008), conceptualizations of key defining factors of corporate responsibility (Garriga & Melé, 2004; Matten & Moon, 2008), reflections on the role of companies in global governance, and the inherent gap that corporate power produces in international governance, particularly related to international human rights law (Alston, 2005; Clapham, 2006), as well as questions relating to the managerial operationalization and implementation of issues pertaining to RBC (Waddock & Rasche, 2012).

While traditionally, conceptualizations of RBC have focused on the social, economic, and environmental dimensions of business activities from a precautionary perspective (‘do no harm’), more recent approaches have explored extended and more pro-active roles of corporate actors in enabling and expanding RBC. This includes the role of business in addressing large-scale societal dilemmas and grievances, commonly referred to as “grand challenges” (George et al., 2016, p. 1880), like global poverty or climate change. This focus on corporations’ duty to help solve pressing societal issues brings to fore the political dimension of RBC (Matten & Crane, 2005; Scherer & Palazzo, 2007), which is particularly evident in cases where expectations of corporate responsibility extend to domains formerly regarded as the exclusive preserve of governments, such as human rights (Arnold, 2010; Cragg, 2012; Wettstein, 2009), or the facilitation of peace in contexts of conflict (Fort, 2009; Katsos & AlKafaji, 2019). As perspectives about the responsibilities of corporate actors have broadened, so has the understanding of RBC: from charitable and philanthropic foci in the 1970s and 1980s, to a focus on addressing, i.e., both preventing and mitigating, the negative impacts of companies’ core business and international value chains in the 1990s, to the promotion of broader principles aimed at improving social and environmental outcomes through multi-sector and multi-stakeholder collaborations both within and beyond a company’s core business (Wettstein, 2018).

Based on this literature, and with particular attention to more recent academic literature and policy debates on corporate responsibility and business ethics more generally, we adopt an *impact-based understanding of RBC* (European Commission, 2011; Ruggie, 2011, 2013) that focuses on the entire value chain of trading companies and their respective impacts on the economic, social, and political dimensions of doing business. Hence, we define RBC broadly as: *the practices and policies of commodity traders and trading companies that aim to responsibly manage value chains by identifying, preventing, and mitigating actual and potential adverse human rights and environmental impacts that are either directly or indirectly associated with their business activities* (Freidberg, 2017a; Martin-Ortega, 2017; Ruggie, 2007, 2013). Beyond identifying, preventing, and mitigating adverse human rights and environmental impacts stemming from or being linked to commodity companies’ business activities, our definition of RBC also comprises conscious efforts by corporate actors to help solve large-scale societal challenges even those that are unrelated to their core activities (George et al., 2016). In this latter sense, RBC can take the form of individual company initiatives or more orchestrated and collaborative efforts among various stakeholder groups (e.g., within the same industry) (Wettstein, 2012).

Importantly for the ensuing literature review, RBC can be formalized through *public* or *private* governance mechanisms that accompany, regulate, and incentivize business activities with a positive contribution to society and the environment (Abbott & Snidal, 2000). *Public* governance mechanisms rely on states developing appropriate laws or incentive-based policies and mechanisms to prompt companies to conduct due diligence and/or implement standards for compliance with their moral, social (including human rights) and environmental responsibilities. *Private* governance mechanisms in turn define standards of conduct for corporate behavior and can be established by companies or industry associations alone or with others, including companies, states, non-governmental organizations, and academia (Freidberg, 2017a; Martin-Ortega, 2017; Ruggie, 2014). Moreover, investors have increasingly shown interest in making decisions based on environmental, social, and governance criteria, or “ESG” (Drempetic et al., 2020, p. 333) for short, which can also drive RBC.

The two mechanisms are based on and aimed at the identification, prevention, and mitigation of tangible adverse social and environmental impacts stemming from or being linked to business activities along the entire value chain, as well as the incentivization and initiation of proactive contributions to addressing grand societal challenges. The distinction between private and public governance mechanisms is reflected in our literature review where we divide the articles in our sample into three themes: (1) industry self-regulation and co-regulatory initiatives (*private* governance mechanisms), (2) government-led regulatory initiatives and policy responses (*public* governance mechanisms), and (3) company-level management strategies impacting RBC practices (*private* governance mechanisms).

## Methodology

While scanning the academic literature on CT and RBC, which was scattered across various academic disciplines, we were struck by the realization that most published articles with significant management implications have been published outside of the business and management field. A significant proportion of relevant research has been published in geography, political science, or sociology journals, to name a few. This prompted us to undertake a multidisciplinary review (Nijs et al., 2014), to synthesize relevant discussions across disciplinary silos. To this end, we applied a three-step systematic review approach: (1) planning the literature review, (2) identification and selection of relevant articles, and (3) analysis of articles (Tranfield et al., 2003).

**Fig. 2:**
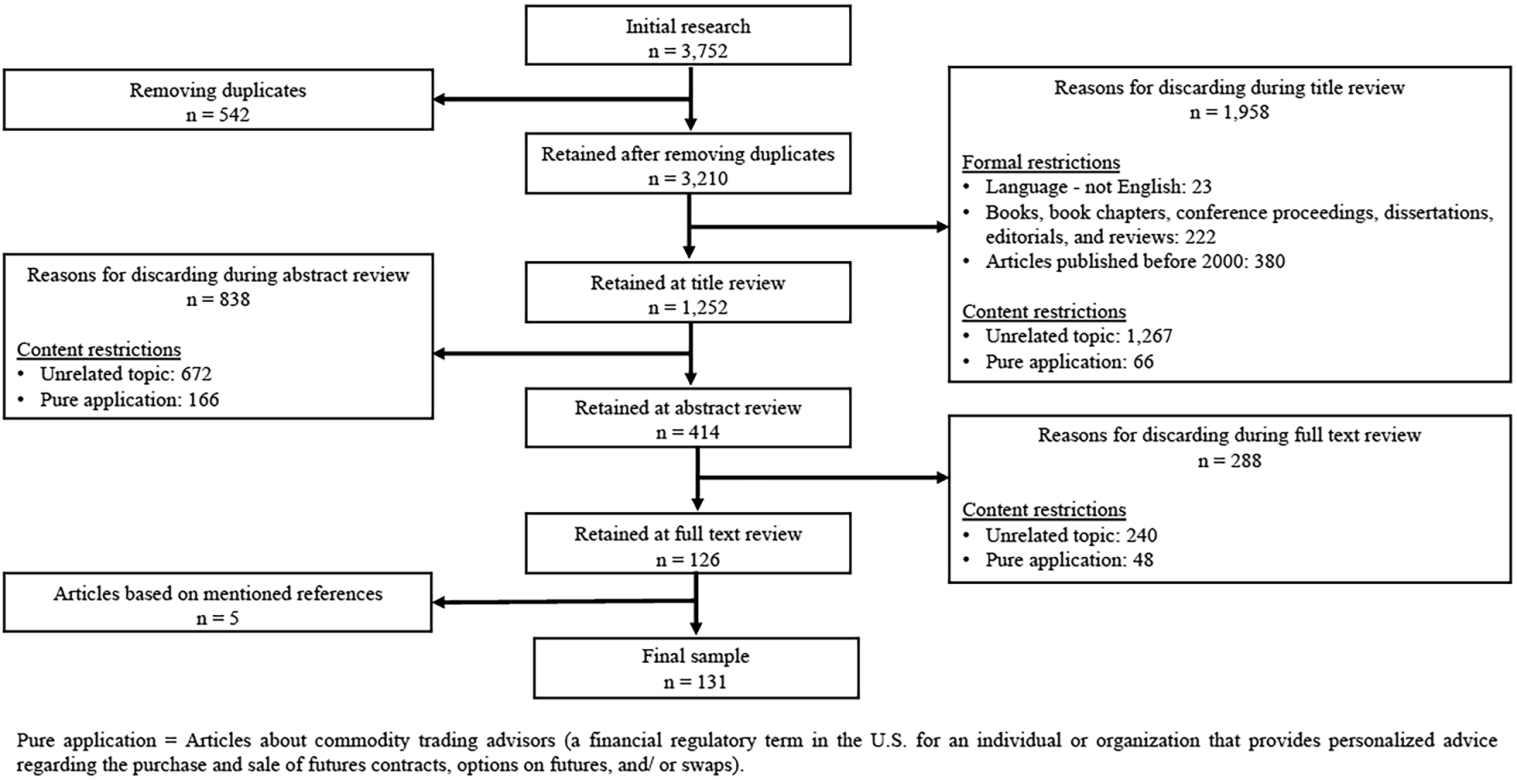
Overview of the identified and included articles

At the *planning stage*, we defined our research goals and questions, identified the time frame of our review, key words guiding the search process, criteria of inclusion and exclusion, as well as our sources of data (journal articles). Based on our team’s knowledge of the RBC debate (in academia and policy), we first scanned general management and business ethics journals^2^ that had already published issues or papers on RBC to identify articles explicitly related to CT (e.g., Academy of Management Journal, Business and Human Rights Journal, Business & Society, Business Ethics Quarterly, Business Ethics: A European Review, Journal of Business Ethics, Journal of International Business Studies, Journal of Management, Journal of Management Studies, Organization Science, and Organization Studies). Limiting our focus to journals from our immediate epistemic community, however, yielded only a limited number of articles, prompting us to expand our search to other scholarly disciplines (notably African, development, energy, environmental, and legal studies, food and agriculture, mineral economics, political science, and sociology). We utilized the following databases for our multidisciplinary search: EBSCO Host, Emerald, JSTOR, Proquest, Sage, Science Direct, Springer, Taylor and Francis, Web of Science, and Wiley. To this end, we identified specific search terms covering a range of topics pertaining to RBC including, human rights, environmental impacts, sustainability, hedging, speculation, illicit financial flows, and corruption. A full list of the identified key words and search string configurations can be found in Table 1. We also searched for different commodity types, as it is not uncommon for authors to focus on the specificities of a single commodity value chain. After several refinements, we considered our sampling approach to be inclusive and comprehensive, and thus consistent with CT’s versatile nature. Focusing exclusively on peer-reviewed journal articles, this search produced an initial sample of 3752 articles from different journals and scholarly domains.The *identification* of relevant articles was set up as an iterative process (see Fig. 2).We first read the abstracts and considered only those articles that *directly aligned with our research interest* (i.e., the relationship between CT and RBC) and that had *immediate managerial implications*, while excluding studies that were not directly related to RBC, such as, contributions focusing on historical aspects or geological perspectives of CT, or detailed commodity extraction processes or technologies without addressing issues pertaining to responsibility. We limited our multidisciplinary review to articles published between 2000 and 2021, since our scanning of the literature revealed that debates on RBC in CT picked up in the early 2000s with a focus on traders’ increasingly prominent role in GVCs (Fold, 2001, 2002; Losch, 2002; Schrage & Ewing, 2005). To be included in our sample, articles were to be published in English with a complete text version. This yielded 414 articles from 150 journals. Next, we further narrowed our selection to articles specifically focusing on CT, specific commodity traders as well as other activities with direct links to the commodity sector such as natural resource extraction or production. Also included were articles on regulation that applied to (some) commodities in general or CT more specifically (including Section 1502 of the US Dodd Frank Act^3^ and the OECD Due Diligence Guidance for Responsible Supply Chains of Minerals from Conflict Affected or High-Risk Areas). Furthermore, we included articles exemplifying the link between physical and paper trading, such as those discussing the social and environmental impact of financialization of commodities. This process yielded a final sample of 131 articles. Table 2 provides an overview of the journals included in the review (and their respective disciplinary focus), as well as the number of relevant papers published therein.

**Table 1:** List of keywords and search strings

First keyword	Second keyword	Third keyword
Responsible business conduct*	Commodity trading* OR Commodity trade* OR Commodity trader*	Human rights* OR Environmental impacts* OR Sustainability* OR Hedging* OR Speculation* OR Illicit financial flows* OR Corruption* OR Metals* OR Minerals* OR Gold* OR Cobalt* OR Copper* OR Agriculture* OR Cocoa* OR Coffee* OR Soy* OR Palm oil* OR Oil* OR Gas* Or Coal*

**Table 2:** Academic journals and their disciplinary and paradigmatic focus

Acdemic journal	Disciplinary focus	No. of articles	Paradigmatic focus	Column 1	Column 2
			Descriptive	Explanatory	Normative
Journal of Modern African Studies	African Studies	3	2	1	
Business and Human Rights Journal	Business and Human Rights	1	1		
Netherlands Quarterly of Human Rights	Business and Human Rights	1			1
Journal of Business Ethics	Business Ethics	3	1	2	
Conflict, Security & Developmemt	Development Studies	1	1		
Development and Change	Development Studies	3	3		
Development Policy Review	Development Studies	2	1		1
Enterprise Development and Microfinance	Development Studies	1	1		
International Development Planning Review	Development Studies	1		1	
International Development Policy	Development Studies	4	2		2
Journal of Peasant Studies	Development Studies	11	10	1	
Third World Quarterly	Development Studies	2	1		1
World Development	Development Studies	1		1	
American Journal of Economics and Sociology	Economics	1		1	
Economy and Society	Economics	1	1		
International Trade Journal	Economics	1	1		
Journal of World Trade	Economics	1			1
Review of Radical Political Economics	Economics	1	1		
Tijdschdrift Voor Economische en Sociale Geografie	Economics	1	1		
Proceedings of the Institution of Civil Engineers—Energy	Engineering	1	1		
Energy Economics	Energy Studies	1		1	
Journal of World Energy Law and Business	Energy Studies	2	1		1
Current Opinion in Environmental Sustainability	Environmental Studies	1	1		
Environment and Planning	Environmental Studies	1	1		
Environment, Development & Sustainability	Environmental Studies	1		1	
Environmental Management	Environmental Studies	2	2		
Forests	Environmental Studies	1	1		
Global Environmental Politics	Environmental Studies	1		1	
International Forestry Review	Environmental Studies	2	1		1
International Journal of Life Cycle Assessment	Environmental Studies	1	1		
Regional Studies	Environmental Studies	1	1		
Sustainability Science	Environmental Studies	1	1		
Water International	Environmental Studies	1	1		
Journal of Agricultural and Environmental Ethics	Ethics	1	1		
European Financial Management	Finance	1		1	
Journal of Applied Corporate Finance	Finance	1	1		
Journal of Financial Risk Management	Finance	1	1		
Physica A: Statistical Mechanics and its Applications	Finance	1	1		
Revista de Economia e Sociologia Rural	Finance	1	1		
Agriculture and Human Values	Food and Agriculture	2	2		
American Journal of Agricultural Economics	Food and Agriculture	1	1		
Cahiers Agricultures	Food and Agriculture	1	1		
Food Security	Food and Agriculture	1		1	
International Journal on Food System Dynamics	Food and Agriculture	1	1		
Journal of Agrarian Change	Food and Agriculture	5	5		
Journal of Agricultural & Food Industrial Organization	Food and Agriculture	1		1	
Journal of Agriculture	Food and Agriculture	1		1	
Annals of the American Association of Geographers	Geography	1	1		
Antipode	Geography	1			1
Economic Geography	Geography	1		1	
Journal of Economic Geography	Geography	2	2		
Tobacco Control	Health	1	1		
Past & Present	History	1	1		
African Journal of Legal Studies	Legal Studies	1	1		
Contemporary Justice Review	Legal Studies	1	1		
Crime, Law and Social Change	Legal Studies	1			1
Global Jurist	Legal Studies	1	1		
Journal of Financial Crime	Legal Studies	1			1
Minnesota Law Review	Legal Studies	2	1	1	
Northwestern Journal of International Law & Business	Legal Studies	1		1	
Social Justice	Legal Studies	1	1		
The International Journal of Human Rights	Legal Studies	1	1		
Business History	Management	1		1	
Business Strategy and the Environment	Management	4	3		1
Competition & Change	Management	1	1		
Cooperation & Conflict	Management	1		1	
Critical Perspectives on International Business	Management	1	1		
Industrial Management & Data Systems	Management	1	1		
International Journal of Engineering and Advanced Technology	Management	1	1		
Journal of International Commerce, Economics and Policy	Management	1	1		
Journal of Management Studies	Management	1		1	
Journal of the Operational Research Society	Management	1		1	
Production, Planning & Control	Management	1			1
Public Relations Review	Management	1			1
Supply Chain Management	Management	1		1	
The Extractive Industries and Society	Mineral Economics	2	1		1
Mineral Economics	Mineral Economics	2	1		1
Resources Policy	Mineral Economics	2		2	
Globalizations	Multidisciplinary	1	1		
Sustainability	Multidisciplinary	1	1		
Global Policy	Political Science	3	1		2
New Political Economy	Political Science	1			1
Problems of Post-Communism	Political Science	1	1		
Review of International Political Economy	Political Science	7	3	3	1
Review of Political Economy	Political Science	1	1		
Sociological Forum	Sociology	1			1
Total		131	83	27	21

In the *analysis* stage, we performed a full text analysis of all articles from our sample with a view toward identifying each paper’s thematic focus, prevailing debates, and gaps. To this end, we inductively coded the articles’ most important aspects and themes (Weber, 1990).

The coding was done manually. Initially, the first two authors picked a sample of ten articles, which were individually coded. They then compared their individual code lists and through a negotiation process, reached consensus on the final code list. This code list (see Table 3 for a summary of the codebook) was then applied to all articles by the five authors. The codebook provided a basis for the team to discuss not just which areas had already been covered in prior research, but also those which have not yet received any or substantial scholarly attention (Van Holt et al., 2021).

**Table 3:** Codebook

Code	Definition
Authors	List of authors
Title	Title of the article
Year	Year of the article
Journal	Name of the journal
Discipline	Disciplinary focus of the journal (i.e. Africa studies, business and human rights, development studies, economics, energy studies, environmental studies, ethics, finance, food and agriculture, geography, health, history, legal studies, management, mineral economics, multidisciplinary, political science, and sociology)
Commodity type	Agricultural commodities (i.e. cocoa, coffee, tea, soy, livestock) or hard commodities, such as energy commodities (gas, oil and coal) and minerals and metals (i.e. 3 TG, aluminium, zinc, copper, cobalt, diamonds)
Research type evaluation	Descriptive, explanatory and normative
Descriptive	Focuses on describing a phenomenon or presenting facts rather than contributing to theory or empirical research
Explanatory	Attempts to explain rather than just describe a phenomenon by focusing on a subject’s constitutive features, processual dynamics, or causal links
Normative	Focuses on making reflective and justified suggestions about how standards, beliefs, behaviours and activities ought to be. It is evaluative and can be based on both philosophical and non-philosophical reasoning
RBC issue	Human rights, environmental impacts, corruption, illicit financial flows, speculation
Value chain stage	Upstream, centre and downstream
Overarching theme	Company-level mechanisms, private global value chain governance schemes, public global value chain governance initiatives

While most descriptive elements of our review are self-explanatory and will be discussed in the findings, two elements need brief explanation here: (1) the value chain segment(s) (an evaluation of whether the article dealt with upstream, midstream, and downstream activities^4^), and (2) the paradigmatic focus (descriptive, explanatory, or normative). The information about the value chain segment allowed us to highlight the extent to which CT was studied in its own right (i.e., as a self-contained commercial activity), or whether it was studied as intimately entwined with other value chain activities taking place more upstream. This information is paramount for understanding whether and to what extent CT is considered as part of a broader and complex value chain.

Second, in analyzing the articles’ paradigmatic focus, the aim was to distinguish articles that are primarily interested in describing or explaining certain aspects of CT (*descriptive* and *explanatory* research, respectively) from those that make a conscious attempt to evaluate and critically scrutinize particular aspects of CT (*normative* research). *Descriptive research* aspires to inform the reader about the state of affairs of the phenomenon under investigation, while mostly remaining at the level of first-order observations. First-order observations rely on cues that are readily available to human perception, and that allow the observer to describe a phenomenon, such as CT, in a matter-of-fact and pre-theoretical type of way. Driven by the objective to accurately and systematically describe a given phenomenon, descriptive research focuses more on the ‘what’ of the phenomenon than the ‘why’ of the research subject. In other words, it ‘describes’ the research subject without covering ‘why’ it happens or why it is in a certain way. Descriptive research often relies on anecdotal evidence based on exemplary cases which is used to define or create tangible knowledge about a phenomenon that might still be relatively nascent. *Explanatory research* in turn attempts to explain, rather than simply describe, why a given phenomenon works in a certain way or why it came into existence to begin with. Explanatory research can be either primarily (a) conceptual (i.e., developing new theories by interpreting and synthesizing exising literature and concepts) or (b) empirical (i.e., producing primary data as the basis for new knowledge and theory). In both cases, the label ‘explanatory’ designates research that is informed by and based upon specific theoretical assumptions and propositions, and that primarily aims to advance theory. The purpose is to provide answers as to how a specific phenomenon is constituted, how its elements or aspects function, or how it is related to other phenomena. Lastly, *normative research* is geared toward making reflective and justified (i.e., value-based) suggestions about how specific aspects ought to be. In other words, it engages with the reasoning underlying the justification of a particular position toward RBC. Normative research can be divided into legal and moral accounts, the latter being characteristic particularly for business ethics scholarship. *Legal studies* use existing hard or soft law frameworks, such as the Kimberly process, an international certification scheme that was established in 2003 to prevent the trade of conflict diamonds, as a normative yardstick for justifying or evaluating specific phenomena related to CT. *Moral accounts,* on the other hand, draw on higher moral principles and values to answer questions about how commodity companies ought to behave, or what behaviors and decisions of corporations are morally acceptable. Such justifications can range from Friedmanian views on profit maximization (Bento et al., 2016) to Kantian perspectives of corporate obligation toward human dignity (Renouard, 2011), to accounts arguing for broader political involvement of corporations (Djelic & Etanchu, 2017). The analysis of the paradigmatic orientation of the articles allows conclusions about whether existing research is primarily oriented towards the descriptive exploration of phenomena pertaining to CT, the development of new explanations and theories of CT-related aspects, or the provision of assessments about as well as recommendations and guidelines for the responsible conduct of CT.

The second part of the full-text reading of the articles in our final sample was done by all five authors and aimed at identifying common themes of commodity traders’ role in value chain governance and related responisibilities. Initially we identified five themes: (1) the role of vertical integration and internal heterogeneity of CT actors, (2) mechanisms to counteract negative human rights/social and environmental impacts of CT including voluntary and mandatory measures, (3) nation states as traders with regards to their financial gains from commodity sales, (4) impact of financialization, and (5) achieving RBC in light of challenges of addressing illicit financial flows in CT. We refined the themes through an iterative process of theoretical reflection within our team, which culminated in the three themes: (1) industry self-regulation and co-regulatory initiatives—including multi-stakeholder and sustainability initiatives aiming at RBC in CT, (2) government-led regulatory initiatives and policy responses on RBC in CT, and (3) company-level management strategies—notably vertical integration and financialization impacting RBC practices.

## Results

In this section we present our findings, starting with the descriptive information of the evolution of scholarly interest as well as the disciplinary and paradigmatic focus of the papers in our sample. This is followed by a discussion of the three identified themes.

### Descriptive findings

#### Evolution of Scholarly Interest

**Fig. 3:**
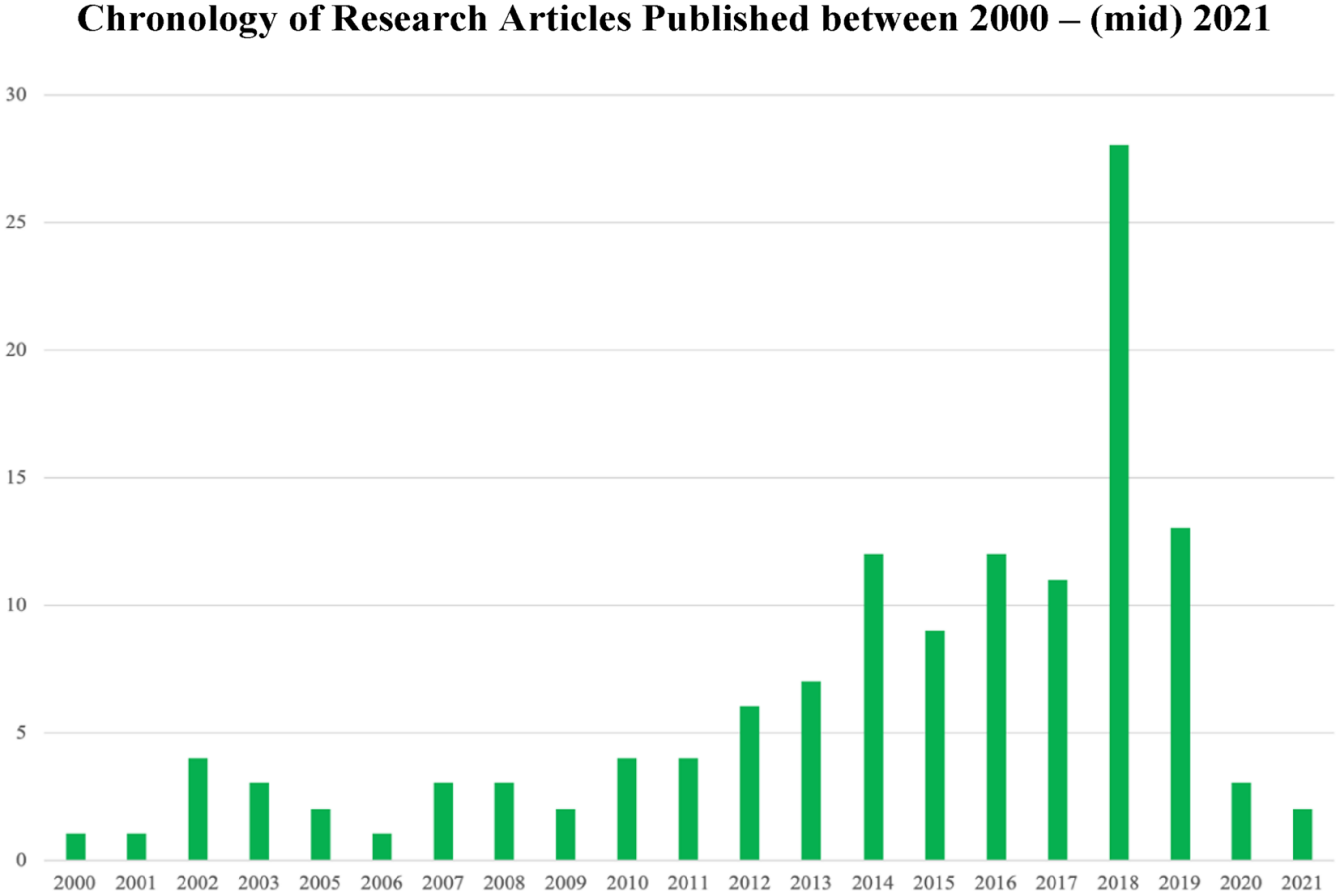
Chronology of research articles published between 2000–(mid) 2021

As seen in Fig. 3, the evolution of scholarly interest in CT was slow at the beginning of our review’s observation period (2000) but has increased in recent years. This is evidenced by the fact that 68% of the articles in our 21-year review were published in the last 7 years (2014–2021).

#### Disciplinary and Paradigmatic Focus

Consistent with the sampling procedure outlined above, Table 1 indicates the multidisciplinary roots of existing CT research, but also suggests that this research topic is still in its infancy, with only one or a few publications in most journals. Although various disciplines engage in research on CT in its relationship with RBC, the majority of articles are published in journals focusing on development studies (26), management (16), environmental studies (13), and food and agriculture (13). Only four articles are published in journals dedicated specifically to (business) ethics (i.e. Journal of Business Ethics: Hira & Ferrie, 2006; Hofmann et al., 2018; Valkila et al., 2010; Journal of Agricultural and Environmental Ethics: Hendrickson & James, 2005).

Table 2 further shows that most articles are descriptive (63%), followed by explanatory (21%), then normative (16%). What can be gleaned from Table 2 is that the majority of articles on RBC in CT is descriptive by adopting a non- or pre-theoretical stance. The relative dominance of descriptive research is not particularly surprising since it is common for new research topics to be initially characterized mainly by anecdotal and definitional research, whereas more explanatory and theory-driven research often only follows as the field becomes more mature (Nicholls, 2010). It is therefore to be expected that with the increasing maturity of research on RBC in CT, the proportion of explanatory research will also increase. Additionally, the overview indicates that only a small number of studies explicitly refer to normative perspectives to acquire clarity on constructs and to justify RBC practices of CT based on legal or moral considerations. To some extent, the relative lack of normative articles might be due to the interdisciplinary nature of our article sample as some of the included subfields do not rely as heavily on legal or moral justifications and evaluations of their object of analysis. Also, normative papers are almost exclusively published in legal and political journals, which account for only 4.5% of the articles analyzed in our review. On the other hand, many of the disciplines identified in this literature review seem generally open to (especially moral) normative perspectives. Taking the example of management studies, which is our own academic home, we can see that moral normative accounts that offer guidance and prescriptions for ethical action and decision-making in various business contexts are regularly published in both specialized business ethics and general management journals. This points immediately to the question of why there is so little (especially moral) normative research on RBC in CT. Irrespective of how the absence is explained, it is imperative for the development and institutionalization of CT research as an independent field of study to develop in the direction not only of greater theory building but also normative reflection (a point we will further elaborate later). The call for more normative research, for which business ethicists are particularly well suited, seems appropriate, especially given that CT is a contentious and contested market activity associated with various adverse social and environmental impacts.

#### Commodity Types

When looking at the commodities dealt with in the analyzed articles, the state of research is skewed toward agricultural commodities with 66% of articles. 3TG (tin, tantalum, tungsten, and gold) and diamonds are given special attention, making up 18% of the articles, while metals make up 9%. Only 7% of articles focus on coal, gas, and oil. Although this is partly speculative, we can surmise that the relative dominance of agricultural commodities in research on CT can be explained by the relative ease of empirical access to the research subject (Salerno, 2017). In contrast, access to actors producing and trading coal, gas, and oil is, as research has shown (Poretti, 2018), more difficult. Research partly supports these speculations as many of the biggest CT companies are leading a rather secretive existence, making it difficult for researchers to access and study them (Bürgi Bonanomi et al., 2015).

#### Value Chain Segmentation

Analyzing the articles according to the different stages of the value chain, i.e., upstream, midstream, and downstream, helps to understand which stages (or combinations thereof) have been the focus of scholarly attention and which ones have received less attention to date. In our sample, the majority of articles focus on the downstream (37%), followed by upstream (31%). A negligible 2% of articles focus exclusively on the midstream. Much of this may have to do with exposure. Indeed, adverse upstream activities, such as human rights violations, environmental impacts, and dismal working conditions are well-documented, and they often have a clear connection to Western brands and consumers downstream. Midstream activities on the other hand are predominantly business-to-business and therefore relatively obscure and off the public radar.

Regarding downstream, some examples of matters discussed include challenges in natural resource revenue management (Asongu et al., 2019; Gillies, 2020; Poretti, 2018), implementation of existing due diligence regulations (Martin-Ortega, 2017; Schütte, 2019) as well as specific instances of wrongdoing by traders (Gillies, 2020; MacManus, 2016; Yaboué & Kaufman, 2018). Since upstream activities concern the sourcing of commodities, the literature discusses the difficulties that smallholder farmers (Amanor, 2012; Ingram, et al., 2018; Millard, 2017; Purcell, 2018; Riisgaard et al., 2010; Wijaya et al., 2018) and miners (Engwicht, 2018; Mullins & Rothe, 2008; Vogel & Musamba, 2017; Vogel & Raeymaekers, 2016) encounter during production and while attempting to access GVCs. Midstream (Dobler & Kesselring, 2019; Grabs & Carodenuto, 2021; Pirrong, 2015) focuses on activities directly related to CT. 12% of the articles adopted a more integrative perspective by looking at both the upstream and downstream (Goldthau & Hughes, 2020; Heron et al., 2018; Wenar, 2013). An additional 8% covered the whole value chain, i.e., upstream, midstream and downstream (Amanor, 2012; Chan & Reiner, 2019; Clapp & Isakson, 2018), and another 10% covered a combination of midstream and upstream or downstream activities (McQuilken, 2016; Sovacool, 2019). This shows, first, that the midstream is grossly understudied. The relative neglect of midstream activities seems problematic, as CT companies could play an important role in the implementation and securing of RBC across entire value chains. Second, it also highlights that few studies on RBC adopt a comprehensive approach to the whole value chain, which seems similarly problematic, as we lack a holistic view of the complex interactions and interdependencies between individual actors in GVC. The conclusions we draw from these two points will be discussed further down.

#### RBC Issues

**Fig. 4:**
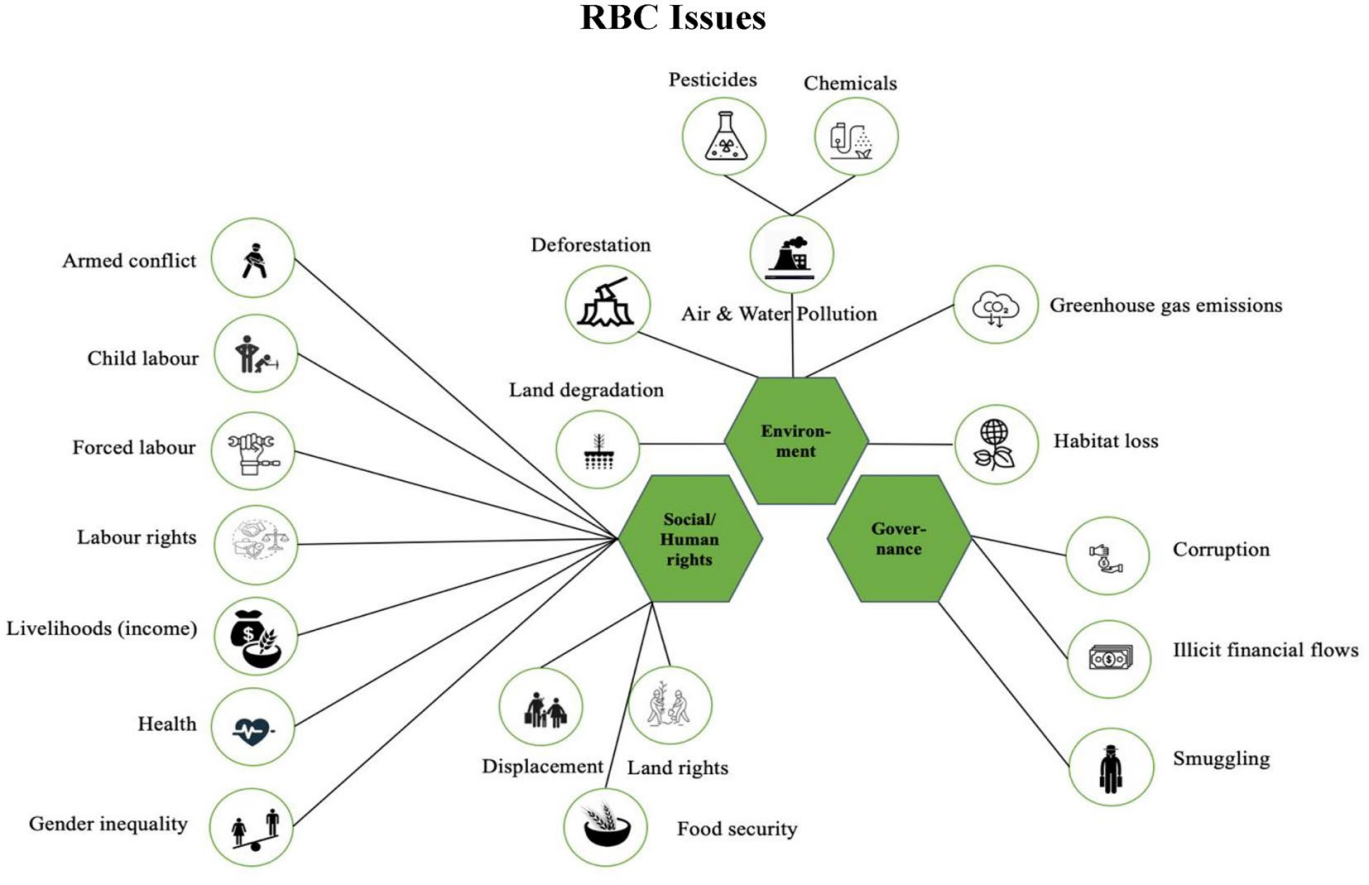
RBC issues

Lastly, we analyzed the articles according to the three concerns in RBC: environmental, social and governance issues^5^ (see Fig. 4). Social issues, which include human rights issues, dominate the literature with 66%. Environmental and governance issues are evenly distributed, each accounting for 17% of the analyzed articles. This distribution of issues may seem surprising at first glance, considering that human rights issues in particular are relatively rarely mentioned in business ethics literature. However, we believe that it is a relatively accurate picture of the specific risks and adverse impacts of the commodity types addressed in the analyzed research papers. Moreover, our analysis suggests that RBC issues of concern are unevenly distributed across the different commodity types. While some are specific to certain commodity types or actors in the value chain, others appear to be of a more transversal nature, occurring across different commodity types. For instance, regarding *agricultural commodities*, we observed a difference of RBC issues between commodities produced by smallholders and commercial farmers. While adverse effects of livelihoods and child labor featured prominently in research on smallholders (Fold, 2002; Grabs & Ponte, 2019; Nelson & Philips, 2018), deforestation, biodiversity (environmental), and displacement (social) featured in research on commercial farmers (Heron et al., 2018; Richardson, 2015; Rodríguez Valle et al., 2018). Regarding *hard commodities,* we observed, among other things, that gas and oil trading is highly susceptible to corruption and illicit financial flows (governance) (Gillies, 2020; Lemaître, 2018; Poretti, 2018). In the case of metals, the main RBC issues covered include illicit financial flows (governance) (Dobler & Kesselring, 2019; Östensson, 2018) and environmental pollution (environmental) (Sovacool, 2019). Research covering 3TG and diamonds in turn identifies a myriad of social (Vogel & Musamba, 2017), environmental (Partzsch, 2018), and governance issues (Wakenge et al., 2018), with armed conflict being a prevalent concern (Bieri & Boli, 2011; Martin-Ortega, 2017; Mullins & Rothe, 2008).

### RBC and CT: Three Overarching Themes

While the previous chapter ended with a tentative overview of RBC issues dealt with in extant research, we will now deepen our analysis by discussing the current state of research on the relationship between CT and RBC. To this end, we group the existing research according to three themes that can guide CT companies toward greater RBC. Two themes were derived directly from our understanding of RBC introduced earlier. These are (1) industry self-regulation and co-regulatory initiatives, and (2) government-led regulatory initiatives and policy responses. The third theme (3) company-level management strategies was inductively derived from the empirical data.

#### Industry Self-Regulation and Co-regulatory Initiatives—Multi-stakeholder and Sustainability Initiatives Aiming at RBC in CT

The literature on RBC generally discusses how private regulatory mechanisms can promote the setting of standards of corporate conduct aimed at advancing responsible business practices, for example, through actions by individual companies, corporate peer-to-peer networks, or collaborations with other, non-corporate actors, such as initiatives with states, civil society organizations, or academia (Freidberg, 2017a; Martin-Ortega, 2017; Ruggie, 2014). The more specific literature on RBC in CT suggests that private governance mechanisms employed by commodity traders can take three forms: (1) participation in existing multi-stakeholder initiatives (MSIs), (2) implementation of consumer-facing company schemes, and (3) implementation of company-level policies.

First, several articles discuss the participation and role of commodity traders in MSIs aimed at RBC (Heron et al., 2018; Nelson & Philips, 2018). MSIs are voluntary mechanisms that bring together various businesses, civil society groups, and governments to foster the implementation of social and environmental standards through governance structures (Ingram et al., 2018; Macdonald, 2007; Millard, 2011). Once these voluntary standards are adopted, participating companies are expected to implement and adhere to them. The literature suggests that commodity traders participate in existing *certification-based* (e.g., Roundtable on Sustainable Palm Oil (RSPO) and Roundtable on Responsible Soy Association (RTRS)) and *principle-based* MSIs (e.g., International Cocoa Initiative). In certification-based MSIs, participating companies are assessed for compliance with the predefined standards after which they are authorized to use the certifier’s label. Principle-based mechanisms, on the other hand, lack a verification or compliance mechanism; instead, they consistently work to improve specific RBC issues based on the voluntary commitments of companies. Some of the most frequently discussed examples of MSIs with traders’ participation include Fairtrade (Hira & Ferrie, 2006; Valkila et al., 2010), the Roundtable on Sustainable Palm Oil (RSPO) (Grabs & Carodenuto, 2021; Richardson, 2015; Schleifer, 2016; Schleifer & Sun, 2018), the Roundtable on Responsible Soy Association (RTRS) (Heron et al., 2018), the Better Cotton Initiative (Snyed, 2014), Bonsucro (Snyed, 2014), the International Cocoa Initiative (Nelson & Phillips, 2018; Schrage & Ewing, 2005; Thorlakson, 2018), the International Tin Supply Initiative (Vogel & Musamba, 2017; Vogel & Raeymaekers, 2016), the Swiss Better Gold Association (McQuilken, 2016), and the Extractive Industries Transparency Initiative (EITI) (Poretti, 2018).

Second, existing studies show that consumer-facing companies (trading mainly in agricultural commodities) heavily rely on traders to implement their sustainability standards, as they often have a better understanding of and relationship with operations in producer countries (Alvarez et al., 2010; Freidberg, 2017a; Hofmann et al., 2018; Ingram et al., 2018). Alvarez et al. (2010) take the example of the Nespresso AAA program for coffee, which Nestlé launched in 2003 and has since been implemented by commodity traders. Through a series of interventions, the AAA program aims at supporting farmers to produce higher quality coffee and at the same time improve their livelihoods and those of their communities, while protecting the environment and addressing several additional RBC concerns along the coffee value chain (Alvarez et al., 2010).

Lastly, the implementation of company-level policies entails commodity traders making ambitious commitments to improve GVCs by launching their own RBC initiatives (Freidberg, 2017b; Thorlakson, 2018). For instance, agricultural commodity traders implement such policies, among other things, through programs that aim to support and empower smallholders through training activities, provision of credit, and other non-financial resource inputs (e.g., seeds and fertilizers) (Deans et al., 2018). These activities bring traders closer to the producer level, thereby enabling collection of farm-level data, which improves traceability and transparency efforts (Freidberg, 2017a; Rueda et al., 2018). More interaction at this level can increase companies’ understanding of stakeholders’ concerns and improve their responses, thus contributing to the promotion of RBC.

Having highlighted the potential of these different governance forms, it is important to keep in mind that industry self-regulation and co-regulatory initiatives (MSIs) may come with their own challenges. Ingram et al. (2018) compare the situation of UTZ, a certified labelling program for various agricultural commodities (which translates to ‘good coffee’), with non-certified cocoa farmers in Ghana and Ivory Coast, and find that certification only has positive effects on farmers’ earnings, crop yields, and livelihoods if they receive a comprehensive bundle of farm equipment and services. The work by Nelson and Philips (2018) further challenges the effectivenss of certification schemes in resolving poverty and child labor issues, arguing that transformative change necessitates “farmer and community political empowerment to drive economic change” (p. 260). Other articles critically reflect on power dynamics in certification schemes as well as existing North–South dynamics and underlying relations of power. McQuilken (2016) assesses the ability of a set of mineral certification initiatives to empower artisanal miners, pointing out that they appear to mostly target the situation of commodity traders and refiners, rather than artisanal miners. Wijaya et al. (2018) explore a multi-stakeholder program funded by public and private actors from the North and aiming at improving farming practices in the Global South, observing tensions between the Northern initiators’ framings of the problem and the divergent framing of the farmers. Schleifer (2016) as well as Schleifer and Sun (2018) observe how specific market conditions shape the local uptake of private governance regimes (specifically the Roundtable on Sustainable Palm Oil), stating that social and environmental standards are still in their infancy and only starting to gain traction.

On the question of governance, the key private or soft law regulation referred to in the literature is the EITI whose primary aim is to increase transparency of government revenues from the hard commodities oil, gas, and minerals. Commodity traders are also part of the initiative, and given the sector’s susceptibility to corruption (Poretti, 2018), this has the potential to curb corruption and other forms of illicit financial flows (IFFs). Whether these issues can be controlled or prevented depends on collaborative disclosure by revenue-receiving states (Lemaître, 2018). Despite the link between corruption and IFFs and the (failed) fulfillment of human rights (United Nations, 2020), few articles have yet addressed this focus (Gillies, 2020; Schlenther, 2016).

In summary, RBC is becoming increasingly important for companies in general, and the literature suggests that commodity traders are seeking to play a positive role either through certification efforts, implementation of their own sustainability commitments, or collaboration with other stakeholders. However, given the various studies questioning the effectiveness of industry self-regulation and co-regulatory initiatives to ensure RBC, this implies that these issues require further attention and critical examination.

#### Government-led Regulatory Initiatives and Policy Responses on RBC in CT

In addition to private governance measures just discussed, government regulation can be another way to encourage companies to move toward RBC. This can be in the form of ‘hard’ law that requires companies to undertake and report on human rights and environmental due diligence, or incentive-based policies that require companies to disclose social and environmental parameters and their approach to RBC (Abbott & Snidal, 2000). The reviewed articles list several domestic and international regulations applicable to the trade of minerals and metals (Martin-Ortega, 2017; Partzsch, 2018; Schütte, 2019; Vogel & Raymaekers, 2016; Voland & Daly, 2018). This mostly includes laws aimed at achieving traceability through due diligence, a process though which companies identify, prevent, and mitigate human rights, environmental, and governance impacts.

Hard law approaches are particularly prominent in the context of conflict minerals such as tin, tantalum, tungsten, and gold (3TG) (Martin-Ortega, 2017; Partzsch, 2018; Schütte, 2019; Vogel & Raymaekers, 2016; Voland & Daly, 2018). Laws in this domain were passed mainly because of resource conflicts rooted in the exploitation of vast natural resources in resource-rich countries. Exemplary in this regard is the Democratic Republic of Congo, where resource extraction incited violence, and grave human rights abuses (Martin-Ortega, 2017; Partzsch, 2018; Schütte, 2019; Vogel & Raymaekers, 2016; Voland & Daly, 2018). Laws that were passed to curb these conflicts include Section 1502 of the U.S. Dodd-Frank Act (2010), the EU Conflict Minerals Regulation (2017),^6^ and The Swiss Ordinance on Due Diligence and Transparency in relation to Minerals and Metals from Conflict-Affected Areas and Child Labour (2021).^7^

There is a burgeoning literature examining the scope and content of these and other regulatory frameworks, comparing elements of different regulations or assessing their respective impact and effectiveness on the ground. Voland and Daly (2018) compare, for example, the US Dodd Frank Act of 2010 (Section 1502), the OECD Due Diligence Guidance for Responsible Supply Chains of Minerals from Conflicted Afflicted and High-Risk Areas of 2011, and the EU Regulation on Conflict Minerals which came into effect in January 2021. The article focuses on the differences and complementarities of these frameworks and concludes that the impact of the regulations on artisanal and small-scale mining (ASM) on the ground remains to be seen. Another insightful example includes Schütte’s (2019) empirical study that uses tin and tantalum trade data from the East-Central African Great Lakes region to assess the negative impact of mandatory due diligence laws on ASM producers. The article finds that the decline in exports immediately following the enactment of the Dodd Frank Act was due to the need for an adjustment period rather than to market access restrictions for ASM producers. Schütte (2019) further notes that the law appears to have levelled the playing field and attracted more traders, but urges for more research on the distribution of revenue along the value chain to understand the parity of cost sharing among the involved actors. Vogel and Raeymaekers’ (2016) ethnographic research on the impact of implementing new laws—such as conflict minerals regulation for ASM in the Democratic Republic of Congo—indicates their potentially negative influence, as they could ostensibly exacerbate existing conflicts rather than mitigating them. This paradoxical finding, which is emblematic of the yet limited research on the effects and effectiveness of hard law initiatives, calls for more granular research aimed at understanding how laws impact mining sites, local communities, and exporting countries at large.

Other researchers have been more pointed in their views on whether the policy objectives pursued through hard law initiatives actually reach their goals (i.e., successfully deal with specific challenges on the ground). Vlaskamp (2019) for one argues that the EU enacted its conflict minerals regulation (based on a due diligence rationale) primarily with the objective of promoting transparency in global supply chains. This strategic approach was driven by the EU’s pursuit of political goals, notably the promotion of peace and enhancement of the country’s security situation, was well as economic goals, including ensuring a consistent supply of raw materials (Vlaskamp, 2019). Partzsch (2018) takes an even more critical view, noting that so long as EU countries as home-states are unwilling to hold their businesses accountable with regards to their conduct abroad, requiring them to address the impacts on the ground, the EU regulation will only have “symbolic normative power” (p. 479).

In summary, while a hard law approach seems sensible given the challenges CT poses, it needs to be kept in mind that the majority of hard laws are enacted in the Global North and apply only to a narrow category of minerals and metals. By implication, current laws leave many commodity types unregulated. Furthermore, the effectiveness of existing laws is neither supported by robust research, nor has it been called into question by critical commentators, so more research is needed in this regard, especially as more laws are continuously passed (European Commission, 2022).

#### Company-Level Management Strategies—Vertical Integration and Financialization Impacting RBC

Our third theme covers company strategies, specifically vertical integration and financialization. These strategies, which are not explicitly related to the literature on RBC, were inductively derived and relate to specific characteristics of CT companies. The reason why they are mentioned here is that they do have very strong implications for RBC. Both vertical integration and financialization have led to a concentration of power by large CT companies. This puts these new powerhouses in a situation in which they become agenda setters also in the realm of RBC. The concentration of actors in CT caused by vertical integration and financialization ultimately creates additional risks and affects (best) practices in the sector’s risk assessment, thus either supporting or weakening companies’ RBC.

Whereas there has been a steady swell of research focusing on the control, organization, and governance of GVCs, some scholars have studied CT companies’ powerful role in GVCs through vertical integration. Through this debate, scholars have responded to the need to broaden our understanding of GVCs (Kano, 2018; Strambach & Surmeier, 2017) by including CT companies as critical actors (Baines & Hager, 2021; Dobler & Kesselring, 2019; Fold, 2002; Grabs & Ponte, 2019; Leguizamón, 2016). For instance, Baines and Hager’s (2021) research hints at CT’s oligopolistic nature by highlighting that the four largest agricultural commodity companies control 75–90% of the global grain trade; the three biggest Swiss-based commodity traders manage half of OPEC’s total oil output; and a single Swiss-based company controls 55% of the worldwide zinc market. Several scholars have used the soy (Heron et al., 2018; Leguizamón, 2016; Oliveira, 2018; Oliveira & Hecht, 2016; Oliveira & Schneider, 2016; Wesz, 2016), cocoa (Fold, 2001, 2002; Losch, 2002; Purcell, 2018; Thorlakson, 2018), and coffee value chains (Grabs & Ponte, 2019; Ponte, 2002; Talbot, 2002) as primary examples of how agricultural commodity traders’ power has grown significantly over time. Vertical integration and power are closely linked, as vertically integrated agricultural traders not only own a large part of the storage, processing, and production infrastructure (downstream), but are also involved in financing activities such as land investments (upstream). A prominent example in this regard is Louis Dreyfus’ Calyx Agro Ltd, a private equity investment vehicle and part of the agricultural merchant and processor Louis Dreyfus Company, that purchases, operates, and sells land in Latin America. Ownership of hedge funds, according to Salerno (2014), has increased agricultural trading companies’ power, making them “more dispersed across various networks and throughout numerous value chains” (p. 1711). Another example is Cargill’s Black River Asset Management, a global hedge fund that manages over $10 billion in assets (Burch & Lawrence, 2009). Scholars have analyzed how the power concentration of agricultural CT companies has been enabled through vertical integration and how this (negatively) affects producers’ situations and labor conditions. Indicative in this regard is Amanor’s (2012) study that uses the examples of the Ivorian cocoa and Ghanaian pineapple industries to show how commodity markets characterized by high concentration (i.e., a few actors dominating the market, to the point of a monopoly situation) are associated with a higher likelihood of smallholder dispossession. Dispossession thereby occurs either through the displacement of struggling farmers or through market struggles that lead to a collapse in farm-gate (producer) prices and increased vulnerability and precariousness among farmers. Metal and oil trading companies have not been excluded from this trend toward vertical integration. They are, among others, acquiring mines, expanding their oil refining and petrol distribution networks, as well as moving into or extending warehousing facilities (Baines & Hager, 2021; Dobler & Kesselring, 2019; Jacobs, 2014). In their article, Dobler and Kesselring (2019) reflect on how Swiss trading companies have used vertical integration to gain more control over the value chain, in the process capturing significant financial benefits from the Zambian copper industry to the detriment of the host country.

The second key driver of the re-configuration of global commodity value chains is financialization. In broad strokes, financialization refers to the growing importance of finance in the global economy and has been typified by the evolution of new financial markets, financial actors, financial institutions, and financial services and products (Epstein, 2005). While the financialization of the globalized economy is still evolving (Sawyer, 2014), the term has great explanatory power in relation to commodities by bringing into view how investors engage in commodity futures contracts as an investment vehicle, and at the same time, traders participate in the futures markets to manage their risks (Clapp, 2014; da Silva et al., 2003; Haigh & Holt, 2000; Newman, 2009; Pirrong, 2015).

Commodity companies have increasingly become big capital lenders in resource-rich countries (Norouzi, 2021). Natural resources are thereby used as collateral for so-called resource-backed loans from trading companies to governments or state-owned businesses (EITI, 2020). The risks of this specific financialization practice are evident in cases such as the Republic of Congo, which has found itself in a severe debt crisis with resource-backed loans representing 70% of the total public external debt (including $1.5 billion in loans coming from two commodity traders) (Norouzi, 2021). Evidence suggests that such loans lead to various additional problems: corruption, lack of public control over public debt, and the risk that the funds are used to finance conflicts (Norouzi, 2021).

The literature further discusses financialization in relation to production. An example is the Mutoshi pilot project on responsible cobalt sourcing in the DRC initiated by Trafigura (a multinational company trading in base metals and energy) (Calvão et al., 2021). Between 1000 and 5000 miners participated in the project, the main aim of which was to formalize artisanal mining and in the process rendering it more responsible through improved controls and safety standards and through the partial mechanization provided through training and material and equipment support. On their part, the miners were required to join a cooperative which negotiated an offtake agreement with a third party which in turn was sold to the company. With limited price guarantees, the miners were exposed to the risk of reduced earnings, while the offtake agreement reduced their collective representation and negotiation capacity. The assessment of the Mutoshi project remains ambivalent. On the one hand, it helped the company in managing reputational risks and securing commodity supply. On the other, the model was akin to employing miners as wageless laborers which extended their vulnerability and insecurity, a process termed by Calvão and colleagues as the “corporate outsourcing of responsibility” (Calvão et al., 2021, p. 7).

In summary, CT companies have become increasingly powerful actors in global commodity value chains through vertical integration and financialization. However, their growing size and influence raises concerns for the promotion of RBC, both internally (how to achieve RBC across highly diversified companies) and externally (RBC agenda setting by CT companies). These issues have yet to be properly addressed by CT scholarship.

## Research Gaps and Avenues for Future Research

As revealed by the foregoing review, research at the intersection of CT and RBC, while providing interesting and relevant insights, is still in its infancy. Thus, our overview of the nascent literature on CT and RBC offers ample opportunities to delineate avenues for future research that can address some of the existing gaps mentioned above (see Table 4 for a schematic agenda for future research). We proceed to outline possible research pathways, and identify concrete research opportunities related to the three themes just discussed.

**Table 4:** Areas for future research on commodity trading and responsible business conduct

Research avenue	Identified gap in existing literature	Illustrative research questions
Normative debate on appropriate standards of RBC and acceptable legal or moral boundaries of business conduct in CT	Justificatory grounds of particular standards and business practices to critically inform discussions on legitimate conduct in CT	• How are RBC practices of CT justified on the basis of legal or moral (including economic) considerations? • By which norms and standards ought RBC practices in CT to be assessed? • Which are the predominant critiques to RBC in CT?
Responsible business conduct in CT from a business and management studies perspective	Research into corporate practice implications of RBC and CT, in particular from a business ethics perspective	• Which practices can inform ethical conduct and create practical knowledge for CT managers? • What does responsible conduct look like at the intersections between CT and other actors along the commodity value chain? • What constitutes effectiveness of RBC practices and how can it be measured?
Corporate governance implications and company-level management strategies for RBC in CT	Company-level policies, strategies, and mechanisms for RBC have largely remained implicit in the reviewed literature on CT	• How can RBC in CT be informed by the large body of research, e.g., on human rights due diligence mechanisms and impact assessments in other sectors? • To what extent are the potential and limitations of due-diligence mechanisms in other sectors mirrored in CT, or how do they differ? • How can the large body of literature on corporate responsibility in the extractive industries inform the practice of vertically integrated CT companies?
Corporate responses to government-led regulatory and policy initiatives on RBC in CT	The majority of research on the regulation of conflict minerals has focused primarily on the extraction of commodities (upstream), while neglecting a focus on CT (midstream)	• How do CT companies endorse and implement regulatory requirements? • What type of regulation would be best suited to address CT’s key position in influencing and shaping RBC across sectors and value chains? • How and to what extent do CT companies pass on and reinforce the pressure generated by regulatory instruments along the entire value chain?
Impact of industry self-regulation and co-regulatory initiatives in CT	Effectiveness of company-level standards and initiatives has not yet been conclusively clarified, such as the role of CT companies within MSIs	• To what extent can industry self-regulation translate, refine, complement, and even operationalize hard law, which is often generic and unspecific in regard to implementation? • How can industry self-regulation and co-regulation in CT achieve beneficial outcomes, potentially in combination with regulatory initiatives? • How can the pervasive threat of ineffectiveness and commodification of industry self-regulation be addressed?
RBC implications deriving from vertical integration and financialization in CT	The increasing trend toward financialization and vertical integration in CT has not yet been addressed from an RBC lens in the literature	• Which parallels and differences in comparison to banking and finance stem from market integration, consolidation, and financialization of CT companies in GVC? • What can we learn from the more mature body of literature on RBC in the finance and banking sector in regard to the progressive financialization of CT? • Does vertical integration increase CT companies’ leverage to become progressive forces in the promotion of RBC along the entire value chain?

To begin, in terms of paradigmatic focus, our analysis revealed that most of the published research articles are descriptive (63%), with a very small amount of normative research (16%). This is, as stated earlier, an evident shortcoming not least with respect to the ongoing controversy around appropriate standards of RBC and acceptable normative (legal or moral) boundaries of business conduct in CT. More normative research focusing on the justificatory grounds of particular standards and business practices is called for to critically inform discussions on what is to be considered responsible conduct in CT. A central concern for future research, therefore, should be to assess and justify RBC practices of CT based on legal or moral considerations. In terms of academic disciplines, our analysis has shown that business and management studies (our own academic home) adopt a minor position in terms of published articles. This marks a missed opportunity to the extent that management research is well placed to create practical knowledge for CT managers, wherefore it could play an important role in transmitting insights about the importance and practical implementation of RBC concerns (Schrempf-Stirling & Van Buren III, 2020; Schrempf-Stirling et al., 2022). In a similar vein business ethics is predestined to offer granular insights into the normative justification of RBC issues just discussed, so greater involvement of business ethicists in CT research would be beneficial in the future. Indeed, with its long tradition on normative explorations on themes and topics relating to RBC, business ethics scholarship seems particularly well-placed to make meaningful and relevant contributions to advance this emerging CT research. Possible foci could include research into the moral justification of existing standards of RBC, such as the Kimberly process, to assess whether they meet the criterion of justificatory adequacy (Korsgaard, 1996). Another important area for future research could be to examine the prevailing criticism of CT as expressed by, for example, the media or advocacy groups, and the extent to which it reflects and is consistent with current debates in business ethics on the corporate responsibility of international companies (e.g., Néron, 2010). In this vein, the pro-active role and responsibility of CT companies in advancing RBC along the value chains across various industries will be of particular interest. Equally, business ethicists could also shed light on the moral principles and normative assumptions that CT actors invoke to justify their actions and counter the accusations and claims of their critics. Such a focus enables us to investigate how CT actors engage in processes of justification by reasoning about what is morally right and wrong. Scholars such as Boltanski and Thévenot (2006), for example, provide a sophisticated conceptual language for distinguishing the specific moral logics or ‘regimes of justification’ different actors invoke when attempting to legitimize their views, and how these different moral frames of reference can either support or cause difficulties in reaching agreement with others during conflict. What is more, future research analyzing the intersections of CT actors and other actors of the commodity value chain would allow for a better understanding of how to address pertinent issues, such as by distributed or shared responsibility (Wettstein, 2015; Young, 2006).

More thematically, we can see that at the company-level, three important questions arise that deserve adequate attention in future research. First, company-level policies, strategies, and mechanisms for RBC have largely remained implicit in the reviewed literature on CT. In other words, issues and questions pertaining to RBC in CT are touched on peripherally rather than tackled head-on. Focused efforts should therefore be made to systematically address and deal with such issues in future research. Here too, business ethics can draw on a large body of scholarship on the organization and implementation of ethics and responsibility initiatives to contribute and advance such operational-level perspectives on RBC in CT. In addition, particularly with regard to corporate human rights impacts, there is a large body of research on the nature and form of human rights due diligence mechanisms and human rights impact assessments. This literature, although not dealing with CT directly, can offer important opportunities for cross-pollination by increasing understanding of both the potential and limitations of due-dilegence meachnisms in general and with regard to particular industries more specifically (e.g., Götzmann, 2016; McCorquodale et al., 2017). Another area for future interest could concern the positive and pro-active influence CT companies can exert in shaping RBC along the entire value chain. In particular, the ability of CT companies to influence and guide the decisions and behaviors of upstream actors, notably extractive companies, remains under-researched. Moreover, the industry restructuring described in conjunction with the third theme (i.e., increasing financialization and vertical integration) should be included in future research on company-level responsibility policies, strategies, and mechanisms. Such processes increase the depth and scope of the CT sector’s responsibilities. In this regard insights from the large body of research on corporate responsibility of mining companies, including in the respective contributions from the business ethics literature (Kemp & Owen, 2022; Kemp et al., 2011; Meyersfeld, 2017; O’Higgins, 2006) could be applicable to vertically integrated CT companies. Similarly, the ongoing financialization of CT increases the pertinence of RBC scholarship of the finance and banking sector, partly also with angles relevant to a businesss ethics audience (De Felice, 2015; Dowell-Jones & Kinley, 2011; Guisande et al., 2023; Macchi & Bernaz, 2021). Both bodies of literature are far more developed on matters of RBC than the available body of CT research and can thus provide important opportunities to rethink and expand responsibilities of CT companies, both conceptually and practically. Third, the aforementioned processes of market integration, consolidation, and financialization affect the power of CT companies in GVCs across various industries. Given that many CT companies have expanded their power, future research should therefore address questions and issues related to how the responsibility of CT companies does not only relate to their core business practices, but in a much broader sense to the transition to a more sustainable and responsible global economy in general.

At the level of public regulatory initiatives, our review has identified a limited amount of robust research on the impact and effectiveness of emerging laws aimed at addressing social (including human rights) and environmental concerns of affected people and communities on the ground (e.g., Martin-Ortega, 2017; Schütte, 2019; Vogel & Raeymaekers, 2016; Voland & Daly, 2018). In this context, little is known about how companies endorse and implement regulatory requirements, apart from what is covered by general compliance with existing reporting requirements. The prominent and often dominant position of CT companies within GVCs suggests that a focus on future research should be on whether and how such companies adopt, translate, and implement public regulation. This seems particularly important for gaining a better understanding of the broader impact of such regulation. By a similar token, it is important to investigate the means by which and the extent to which CT companies pass on and reinforce the pressure generated by regulatory instruments along the entire value chain. As an obvious candidate to start, we see value in future research that takes a closer look at existing regulation in the area of conflict minerals. However, much of the research on the regulation of conflict minerals has focused primarily on the extraction of commodities (upstream) rather than specifically on their trade (midstream). For states, the critical positioning of CT companies within GVCs raises the question of whether a sectoral approach to regulating cross-border activity is judicious, and if so, what type of regulation would be appropriate to address CT’s key position in influencing and shaping RBC across sectors and value chains. Future research on this issue is urgently needed considering the general reluctance of governments to engage in sectoral regulation and in regulation of commodities other than conflict minerals and metals.

At the level of industry self-regulation and co-regulatory initiatives, the effectiveness of corresponding standards and initiatives has not yet been conclusively clarified. Also, there is a paucity of research focusing on the interplay between industry self-regulation and co-regulatory initiatives and hard law. Regarding the CT sector, it seems fair to assume that industry self-regulation can complement hard law in several ways. For example, industry self-regulation can translate, refine, complement, and even operationalize hard law, which is often generic, i.e., not providing much-needed granular guidance for practical implementation. Furthermore, industry self-regulation can elevate hard law, which is mostly bound to the domestic realm, to a transnational or global level. Against this backdrop, it is worth assessing the degree to which private and public governance mechanisms in CT can be combined in synergistic ways (Poretti, 2015). The value of such a conjoint or polycentric perspective is predicated on the realization that isolated—public or private—governance accounts may not yield the desired (RBC) effects and outcomes. Indeed, prior research has shown that mere voluntary self-regulation and “Westernized” private governance by companies are not able to fully tackle root causes of prevailing social and environmental issues on the ground (Poretti, 2018). Future research should therefore address the pervasive threat of ineffectiveness and commodification of industry self-regulation, while providing a deeper understanding of whether and how such governance initiatives can actually achieve beneficial outcomes (Gardner et al., 2019). A second area of future research at the level of industry self- or co-regulation concerns the role of CT companies within MSIs. While there is a growing body of literature on the purpose, governance, and legitimacy of such initiatives in general, and from a business ethics perspective in particular, the role and participation of CT companies in MSIs is little explored. Future research in this domain is needed not least because of the aforementioned prominent position and preeminent power of CT companies in GVCs and the resulting potential to develop the industry in the direction of greater RBC. In the same vein, there is need for more research focusing on the intersection of corruption, IFFs, and human rights and the effectiveness of the existing private and public governance mechanisms.

## Conclusions

This review was prompted by the need to better understand the current state of research on RBC with respect to CT. The role and power of CT companies in GVCs is pivotal and constantly growing. Alongside this growth, concerns are being raised about the sector’s adverse social (including human rights), environmental, and governance impacts. Thus, our review focused on how CT companies are related to and can potentially foster RBC, revealing three themes along which discussions on this issue are happening and could be further developed: (1) industry self-regulation and co-regulatory initiatives, (2) government-led regulatory initiatives and policy responses, and (3) company-level management strategies*.* The first two represent common mechanisms that have been used to regulate RBC in CT, whereas the latter are typical business strategies of CT companies. Since the literature on this topic is still in its early stages, our literature review has highlighted several research gaps and suggested ways in which future research, particularly as it pertains to business ethics, could address them.

To conclude, given the increasing call for the sustainable and responsible transformation of the global economy, in which CT companies are implied as exceptionally powerful actors, a stronger focus on how CT companies implement RBC is necessary.

## Footnotes

1 Flat price risk is the change in the absolute or benchmark price of a particular commodity (Pirrong, 2014).

2 We acknowledge the publication of several influential books about CT (such as, for example, Blas & Farchy, 2021; Erklärung von Bern, 2011; Haller, 2019), but our review is solely based on academic journals.

3 The law, which was enacted in 2010, includes Sect. 1502 requiring publicly listed companies to issue statements on responsible mining specific to “conflict minerals” – tin, tungsten, tantalum, and gold in their supply their supply chains (U.S. Securities and Exchange Commission, 2017).

4 Upstream activities consist of production or extraction, transportation, and warehousing; midstream activities refer to transforming raw commodities through refining and processing, as well as storage and transportation; and downstream activities include manufacturing and retailing (Adapted from Swiss Federal Department of Foreign Affairs & Swiss State Secretariat for Economic Affairs, 2018, p. 8).

5 RBC criteria or concerns refer to environmental, social/human rights and governance considerations from the perspective of risks to the society. Not to be confused with the ESG discourse which refers to the same concerns but from a company’s internal perspective.

6 The EU Conflict Minerals Regulation was published in 2017 but came into effect in 2021 (European Commission, 2021).

7 The Swiss Ordinance on Due Diligence and Transparency in relation to Minerals and Metals from Conflict-Affected Areas and Child Labour was published in December 2021 and came into effect in January 2022 (Fedlex, 2022).
